# One-Step Surface-Treatment Reagent (35% 3-O-Ethyl-l-ascorbic Acid Plus 50% Citric Acid Solution) Restores the Shear Bond Strength of Metal Brackets Bonded to Bleached Human Enamel: An In Vitro Study

**DOI:** 10.3390/dj11050110

**Published:** 2023-04-24

**Authors:** Pichanee Saeoweiang, Thanit Charoenrat, Chanat Aonbangkhen, Pattraporn Chobpradit, Paiboon Techalertpaisarn

**Affiliations:** 1 Department of Orthodontics, Chulalongkorn University, 34 Henri-Dunant Road, Wangmai, Pathumwan, Bangkok 10330, Thailand; 2 Center of Excellence in Natural Products Chemistry, Department of Chemistry, Faculty of Science, Chulalongkorn University, Bangkok 10330, Thailand; 3 Department of Chemistry, Faculty of Science, Chulalongkorn University, Bangkok 10330, Thailand; p.chobpradit@gmail.com

**Keywords:** bleaching, antioxidant, ethyl ascorbic acid, shear bond strength, brackets

## Abstract

This study investigates how a new substance, composed of ethyl ascorbic acid and citric acid, affects the shear bond strength (SBS) of metal brackets when bonded to bleached teeth. Forty maxillary premolar teeth were used and randomly placed into four groups (*n* = 10): the control group did not undergo bleaching; the remaining groups underwent bleached using 35% hydrogen peroxide. In group A, 37% phosphoric acid was applied after bleaching. In group B, 10% sodium ascorbate was used for ten minutes before 37% phosphoric acid. In group C, 35%3-O-ethyl-l-ascorbic acid plus 50% citric acid solution (35EA/50CA) was applied for 5 min. The subgroups were bonded immediately after bleaching. The SBS was determined with a universal testing machine and analyzed using one-way ANOVA and then Tukey’s HSD tests. Adhesive remnant index (ARI) scores were determined with a stereomicroscope and analyzed with a chi-squared test. The significance level was 0.05. Group C demonstrated significantly higher SBS values than group A (*p* < 0.001), but was not significantly different than the control group or group C (*p* > 0.05). The ARI scores were significantly different among the groups (*p* < 0.001). In conclusion, enamel surface treatment using 35EA/50CA improved the reduced SBS to an acceptable clinical level and reduced the clinical chair time.

## 1. Introduction

Hydrogen peroxide is a widely used agent for bleaching teeth [1]. Several studies demonstrated that orthodontic patients who required tooth whitening prior to aligning their teeth achieved a more satisfactory outcome [2,3]. Krug and Green reported that the patient satisfaction rate increased by 90% when whitening was incorporated in orthodontic treatment [4]. Orthodontists often find that, in addition to their overall appearance, their patients are unhappy about their tooth color. Although using in-office tooth whitening can encourage patients to undergo treatment or the need for further intervention [5], tooth whitening is not mandatory for orthodontic treatment. It is considered a voluntary approach, depending on patient satisfaction in terms of esthetics. However, after tooth whitening, free radical species from bleaching agents reduce the shear bond strength (SBS) between the bracket and enamel by inhibiting resin polymerization and penetration, as well as decreasing the resin tags’ quality in the etched enamel [6,7]. Furthermore, using a bleaching agent can change the enamel surface, which potentially decreases the metal brackets’ adhesive strength [8]. To regain shear bond strength, it has been suggested to wait for 1–2 weeks before performing bonding after bleaching [9,10]. An in vivo study indicated that using 38% hydrogen peroxide bleach led to an increased bracket bonding failure rate; however, this rate was reduced when the bonding procedure was delayed for 2–3 weeks. Postponing the bonding procedure for 1–2 weeks after bleaching was recommended to restore shear bond strength [11].

To avoid a waiting period, antioxidants have been suggested as a possible solution. One of the most commonly used antioxidant agents in clinical and research studies is sodium ascorbate, which is the sodium salt derived from ascorbic acid. This derivative is a widely used antioxidant to regain the post-bleaching SBS after bleaching without postponing the bonding visit, both in vitro [12,13,14,15] and clinically [16]. Several studies applied 10% sodium ascorbate for 10 min as the gold standard for reducing the shear bond strength [12,13,14,15]. Despite its potential benefits, using sodium ascorbate for bonding requires an extra etching procedure with phosphoric acid [15,17,18,19,20], which conflicts with the preference for rapid treatment among patients at present. Moreover, sodium ascorbate has a short shelf life and is highly unstable, and rapidly oxidizes after being exposed to the air [21,22]. Using a combination of antioxidant and acid etching solutions is a promising approach to reduce the total working time and improve the clinical benefits. However, sodium ascorbate’s instability in the presence of water and oxygen, as well as its neutral pH, make it unsuitable to use in conjunction with acidic solutions [21,23]. Therefore, it is necessary to explore alternative derivatives of ascorbic acid and etchants that are better suited for this purpose.

An ascorbic acid derivative called 3-O-ethyl-l-ascorbic acid, containing an ethyl group at the third carbon position, is extensively used in the cosmetic industry as an antioxidant and anti-aging ingredient [24,25]. This structure in 3-O-ethyl-l-ascorbic acid prevents the ionization of the 3-OH group, which consequently inhibits the oxidation of the molecule [26,27]. 3-O-ethyl-l-ascorbic acid is markedly more resistant to heat and light than ascorbic acids [28]. Its stability is also influenced by the pH level of the solution; however, if the pH is below five, it remains stable. Additionally, the ability of 3-O-ethyl-l-ascorbic acid to counteract free radicals is higher when used at higher concentrations [27,28]. Combining 3-O-ethyl-l-ascorbic acid with an acid-conditioning agent is considered appropriate for enamel etching. Although phosphoric acid is a common choice for bracket bonding, its short application time of 15 to 30 s [29,30] is not compatible with the longer working time required for applying an antioxidant. The prolonged use of 37% phosphoric acid can result in excessive enamel etching and tissue loss [31,32], making it unsuitable for combining with antioxidants. Therefore, a weak acid with a longer working time is needed as an alternative to phosphoric acid for etching enamel while simultaneously providing antioxidation. Citric acid was first introduced for use as an acid etching agent in 1971 [33]. At present, it is commonly used in clinical practice for dental etching and root canal irrigation [34,35,36]. A honeycomb etching pattern was achieved when applying 50% citric acid to enamel for 5 min and the optimum shear bond strength was achieved when applying 50% citric acid for 3 min [33]. Because the application time of the citric acid was long enough for antioxidation to occur, we decided to combine them as a one-step surface treatment.

However, the effect of the one-step surface treatment for teeth bleaching the SBS between the metallic bracket and bleached enamel has not been evaluated. Thus, the objective of this study is to determine the effect of the one-step surface-treatment reagent that combines citric and 3-O-ethyl-l-ascorbic acids as an antioxidant on the 24 h SBS of metallic brackets bonded to bleached human teeth with a composite resin adhesive. The null hypothesis is that the SBS between the control and bleached groups among different surface-treatment protocols are not significantly different.

## 2. Materials and Methods

The Human Research Ethics Committee of the Faculty of Dentistry, Chulalongkorn University approved the study protocol (HREC-DCU 2020-113).

### 2.1. Tooth Sample Preparation

The sample size calculation was performed using the G*Power 3.1.9.7 program. The calculation indicated that 28 teeth were required. However, 40 teeth were utilized in the study. The inclusion criteria comprised human maxillary premolars extracted for orthodontic reasons from 17–30-year-old patients with intact buccal enamel surfaces and who had not undergone any chemical treatments, such as peroxide derivatives, acids, bonding agent, vanish, or other types of bleaching. Any teeth with restorations, caries, cracks on the buccal surfaces, hypoplastic areas, fluorosis, or any enamel structure abnormalities were excluded.

A one-step surface-treatment reagent was prepared by mixing 0.35 g of 3-O-ethyl-l-ascorbic acid (EA) (solid, TCI, Tokyo, Japan) with 0.5 g of citric acid (CA) (solid, Carlo Erba Reagents S.A.S., Barcelona, Spain) in 1 mL of distilled water at 40 °C. The final concentration of the reagent was calculated by dividing the mass of the solute by the final solution volume, assuming that the final solution was approximately 1 mL. Based on this calculation, the approximate concentration of the one-step surface-treatment reagent was 35% for 3-O-ethyl-l-ascorbic acid and 50% for citric acid (%*w*/*v*).

The teeth were disinfected in a 10% formalin solution for 2 weeks. The periodontal tissue and debris were removed, and the enamel was polished with fluoride-free pumice paste. The roots were removed 2 mm below the cemento-enamel junction. The crowns were stored in 37 °C fluoride-free artificial saliva for 7 d before starting the experiment. A total of 40 teeth were randomly allocated to 4 subgroups:

Control group (*n* = 10): 37% phosphoric acid on unbleached teeth for 15 s.

Group A (*n* = 10): 37% phosphoric acid on bleached teeth for 15 s.

Group B (*n* = 10): 10% sodium ascorbate treatment for 10 min on bleached teeth follow by 37% phosphoric acid for 15 s.

Group C (*n* = 10): 35% 3-O-ethyl-l-ascorbic acid plus 50% citric acid solution on bleached teeth for 5 min.

### 2.2. Bleaching and Surface Treatment

The control group’s teeth were not treated and stored in artificial saliva for 7 days prior to bonding. In the 3 experimental subgroups (A, B, and C), the enamel buccal surfaces were polished using fluoride-free pumice powder with a brush attached to a slow-speed handpiece. The teeth were rinsed and dried using a triple syringe, followed by bleaching.

In the control group, 10 teeth were treated with 37% phosphoric acid (Ormco^®^, Orange, CA, USA). The etched surface was 1 mm wider than the bracket base along every margin. The solution was applied using a disposable applicator and a continuous movement for 15 s and rinsed for 30 s and dried with compressed air (oil-free) for 10 s (Table 1). The metal brackets were immediately bonded to the tooth’s surface.

The 35% Hydrogen Peroxide gel (Pola Office^®^, SDI, Bayswater, VA, Australia) was used as the bleaching reagent for the 30 teeth in the remaining subgroups (A, B, and C). The bleaching gel was applied as recommended by the manufacturer. The bleaching gel was applied for 4 sessions (8 min per session, i.e., 32 min in total). Then, the enamel surfaces were rinsed with water (30 s) and air-dried (10 s) (Table 1).

In group B, after bleaching, the teeth’s buccal surfaces were treated using 10% sodium ascorbate with a disposable applicator with a continuous movement for ten minutes, then rinsed in water (30 s), air-dried (10 s), and the tooth’s buccal surface was etched, as described for the control group. In group C, the tooth’s buccal surface was treated with 35EA/50CA under a continuous movement for five minutes, rinsed (thirty sec), and dried (ten sec) with compressed air (oil-free) (Table 1).

### 2.3. Bonding Procedure

**Figure 1:**
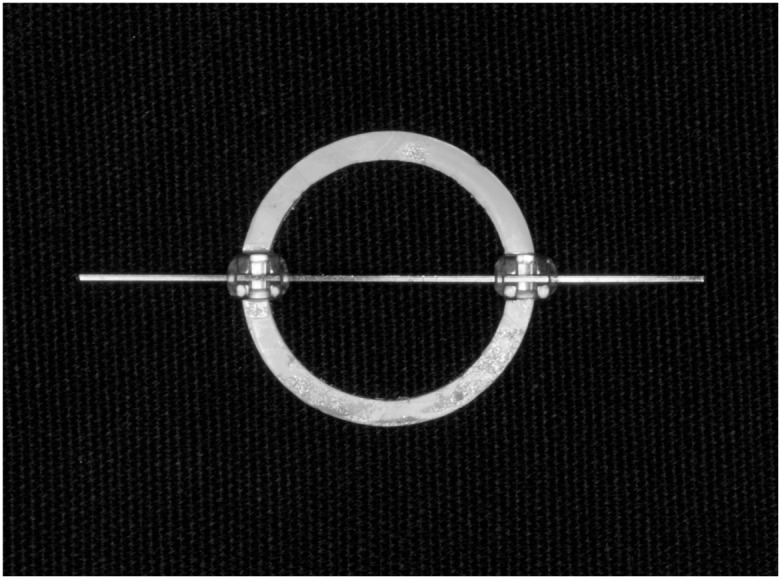
The bonding index was created by attaching two upper premolar brackets to the opposing rims of a PVC pipe so that the universal machine’s blade and bracket margin were parallel.

The bonding procedure was conducted immediately post-bleaching for group A and immediately after surface treatment for groups B and C. The etched surfaces were primed with a TransbondTM XT primer (3M Unitek, St. Paul, MN, USA), then TransbondTM PLUS adhesive (3M Unitek, St. Paul, MN, USA) was placed on the 0.018″ × 0.025″ slot bracket base (Omi arch^®^ Roth type, TOMY, Fuchu-city, Tokyo, Japan) per the manufacturer’s directions. Each bracket was pressed on the enamel surface using a 100 g force (measured by a Dontrix gauge (Orthopli, Philadelphia, PA, USA)). To ensure complete resin filling under the bracket base, excess composite was observed at each margin. The excess composite was removed with an amalgam carver. The adhesive was light-cured for 40 s with an Ortho curing light (Mini LED SATELEC^®^, Acteon, Mount Laurel, NJ, USA) at an intensity of 2000 mW/cm^2^. The curing light was calibrated before being used on each group. A radiometer (DEMETRON, SDS Kerr, Orange, CA, USA) was used to test the curing light prior to the bonding procedures, and this was repeated twice. A bonding index was used for embedding the tooth in an acrylic block so that the universal machine’s blade and bracket margin were parallel (Figure 1). The bonding index was created by attaching two upper premolar brackets to the opposite sides of a PVC pipe using a 0.018″ × 0.025″ rectangular stainless-steel wire. The wire was inserted into the slot of the specimen’s bracket, aligned with the guiding index, and secured to the bracket using elastomeric rings. The specimen was embedded with the palatal cusp in an acrylic block and left undisturbed for 1 h to allow the acrylic to fully set. The specimens were maintained in 37 °C artificial saliva for 24 h before SBS testing.

### 2.4. Enamel Shear Bond Strength (SBS) Test and Adhesive Remnant Index (ARI) Scores

A universal testing machine (EZ-S, SHIMADZU, Nakagyo-ku, Kyoto, Japan) with a load cell of 500 N was used for the SBS assay. The test was performed at a 1 mm/min crosshead speed, in an occluso-gingival direction parallel to the height of the contour. The blade was positioned at the interface between the tooth’s surface and bracket base. The machine recorded the shear force in newtons (Ns). The SBS (MPa) was determined by dividing the shear force by the area of the bracket base. The rectangular shape of the bracket base was measured at its width and height using a digital vernier caliper. To determine the bracket base area, the width and height measurements were multiplied, resulting in a calculated bracket base area of 12.28 mm^3^.

Following the debonding procedure, each tooth’s enamel surface was examined to determine the fracture pattern. The adhesive remnant index (ARI) scores were identified using a stereo microscope (SZ 61, Olympus, Kamiina-gun, Nagano, Japan) at 20X. The teeth were evaluated by the same observer, and the failures were categorized according to the ARI scores:

Score 0: No composite remnants on the enamel surface (failure between the adhesive and enamel).

Score 1: Less than 50% of the composite remaining on the enamel surface.

Score 2: More than 50% of the composite remaining on the enamel surface.

Score 3: The entire composite remained on the enamel surface with an impression of the bracket base on the composite, i.e., adhesive failure.

### 2.5. Statistical Analysis

The results were analyzed using the SPSS program (SPSS version 22, statistical software). The data’s normality was determined using the Kolmogorov–Smirnov test. One-way analysis of variance (ANOVA) was used for identifying significant differences in SBS among the groups. Tukey’s HSD test was used for post hoc multiple comparisons. The chi-squared test was utilized for analyzing the differences in the ARI scores among the test groups. A significance level of 0.05 was used for all comparisons.

## 3. Results

The Kolmogorov–Smirnov test revealed that the data had a normal distribution (*p* > 0.05). The SBS results are presented in Table 2. The control group demonstrated the highest SBS score (19.81 ± 4.15 MPa), followed by groups C (15.84 ± 2.87 MPa) and B (12.09 ± 2.67 MPa). Group A had the lowest SBS score (8.55 ± 3.670 MPa).

The one-way ANOVA results indicate that the SBS scores among the groups are significantly different (*p* < 0.001) (Table 2). Tukey’s HSD test revealed that the 35% hydrogen peroxide-bleached teeth without surface treatment (group A) had a significantly reduced bracket SBS (*p* < 0.001). Treating the enamel surface with 35% 3-O-ethyl-l-ascorbic acid plus 50% citric acid solutions (group C) significantly increased the shear bond strength compared with the bleached teeth without surface treatment (group A) (*p* < 0.001). The SBS was not significantly different compared with the control group (*p* > 0.05) and was not significantly different compared with the surface treatment with 10% sodium ascorbate solution (group B) (*p* > 0.05). Although applying 10% sodium ascorbate solution followed by 37% phosphoric acid etching (group B) increased the SBS compared with the bleached teeth without surface treatment (group A), the mean value was not significantly different (*p* > 0.05). The shear bond strength significantly decreased when applying the 10% sodium ascorbate solution followed by the 37% phosphoric acid etching after bleaching for group B compared to the control group (*p* < 0.001).

The enamel surface evaluation after bracket debonding revealed that significant differences existed in the ARI scores among the four groups. The ARI scores in the bleaching groups (A, B, and C) were typically 0. In contrast, score 2 was most commonly identified in the control-group specimens (Table 3).

## 4. Discussion

Using hydrogen peroxide as a bleaching reagent produces free radicals and reactive oxygen species. Free radicals are any molecules that have one or more unpaired electrons resulting in high reactivity. These molecules can interact with the pigments present in the tooth structures that have a high number of electrons, causing the degradation of larger pigmented molecules into smaller molecules with less pigmentation [37].

The present study investigated the effect of 35% 3-O-ethyl-l-ascorbic acid/50% citric acid, a new reagent for surface treatment, on the SBS of metal brackets bonded to bleached human teeth with a composite resin adhesive. We observed that the SBS was significantly different among the groups. Therefore, the null hypothesis was rejected.

Our results reveal that using 35% hydrogen peroxide in office for tooth bleaching significantly decreased the shear bond strength after immediate bonding, which was similar to the previous studies [5,13,15,38]. These results may be achieved by free radicals that interfered with adhesive system infiltration and inhibited their polymerization [39].

The 10% sodium ascorbate pretreatment group demonstrated an increase in immediate SBS compared with the untreated bleached enamel; however, the difference was not significant. Moreover, we observed that using 10% sodium ascorbate as a pretreatment solution significantly lowered the shear bond strength compared with non-bleaching. These results imply that 10% sodium ascorbate pretreatment may not be suitable for restoring the SBS. Our results differ from the findings presented in previous studies [12,13,14,17,20], which reported a significant improvement in the shear bond strength. The different outcomes might be due to the different methods used: using carbamide peroxide as a bleaching agent [12,13], using 35% peroxide for a shorter treatment time, and single applications of a bleaching agent, which caused less residual free-radical species compared with our study, which had four application cycles, as specified by the manufacturer [14,17]. Coppla et al. also observed that the enamel bond strength significantly increased when 35% sodium ascorbate rather than 10% sodium ascorbate was applied to 35% hydrogen peroxide-bleached teeth [40]. The in vitro studies demonstrated that using an antioxidant solved this problem [40,41,42].

Our results indicate that using a one-step surface-treatment reagent, i.e., 35% 3-O-ethyl-l-ascorbic and 50% citric acids, resulted in a significant increase in immediate SBS compared with the bleaching group without surface treatment. Furthermore, the SBS of the treated group was not significantly different compared with the unbleached control group or the group receiving a 10% sodium ascorbic surface treatment. Our results suggest that the one-step surface-treatment reagent could be a viable option for restoring SBS while reducing the treatment time and steps. The possible mechanism for restoring the SBS is that etching the enamel with a solution containing citric and 3-O-ethyl-l-ascorbic acids effectively neutralizes reactive oxygen species. The reason why the 5 min surface treatment resulted in a good shear bond strength comparable to the control, and not significantly longer than 10 min with the 10% sodium ascorbate solution, was that the reaction reached its maximum value after approximately 1 min, followed by a significant reduction. Our study results align with those of Freire et al., who revealed that the reduction reaction kinetics between sodium ascorbate and hydrogen peroxide were dose-dependent and the antioxidant efficiently neutralized the oxidizing agent in 5 min [43]. Furthermore, another study by Freire et al. demonstrated that increasing the concentration of the antioxidant and using a reduced treatment time restored the reduced shear bond strength [44]. Thus, we increased the concentration of 3-O-ethyl-l-ascorbic acid as an antioxidant agent to 35% in the present study.

Removing the resin on the enamel surface after bracket debonding is clinically favorable because it can minimize the damage incurred during debonding. ARI scores are commonly used to assess the bracket debonding interface [45]. The ARI scores of the different bleaching and etchant conditions were significantly different among the groups. Score 0 was predominant in the bleached groups. These results indicate that bond failure typically occurs between the tooth and adhesive. Corresponding to the previous studies, the non-bleached-teeth ARI patterns were different from the other bleached-teeth groups [13,46]. These results might be because the free radicals were not totally removed by the antioxidant. This was evident from the increase in SBS, which was lower was, but not significantly different from the unbleached teeth.

There are several in vitro studies that used different natural antioxidants, including pine bark extract, green tea extract, grape seed extract, and lycopene, as a surface treatment following tooth bleaching and before the bonding process [21,22,23]. These studies observed that natural antioxidants enhanced the bond strength to bleached enamel; however, these antioxidants required an application time longer than 10 min [23]. Moreover, it is always required to etch the enamel using phosphoric acid before bonding, which makes the method more complicated. However, the results of the present investigation indicate that the antioxidant application time can be reduced to 5 min, reducing the process to washing, blowing, and applying the phosphoric acid while still increasing the shear bond strength of the bleached enamel.

One limitation of this study was that it was performed in vitro, and therefore may not fully reflect the clinical condition. Future research should investigate the in vivo effectiveness of the new reagent because, at present, there is no evidence regarding the survival rate of brackets bonded to bleached teeth that have undergone antioxidant surface treatment. However, SBS can be used as an indicator of the bracket survival rate [47]. Another limitation was that this study only investigated the SBS at 24 h and did not examine the long-term SBS. However, Carlos et al. demonstrated that the shear bond strength after bleaching was not affected by the artificial aging of the materials using thermocycling [48]. Lastly, the long-term stability of the 35% 3-O-ethyl-l-ascorbic acid reagent should be evaluated before it can be used as a ready-to-use product.

The clinical application of this study is that bleaching with 35% hydrogen peroxide significantly decreased the shear bond strength, and surface treatment with 10% sodium ascorbate prior to bonding of in-office bleaching with 35% hydrogen peroxide did not significantly improve the shear bond strength. The 35% 3-O-ethyl-l-ascorbic acid/50% citric acid solution can regain the decreased SBS. Our new reagent can be used as an option rather than 10% sodium ascorbate to regain the decreased SBS and reduce the treatment steps and clinical chair time.

## 5. Conclusions

In conclusion, our study determined that bleaching teeth with 35% hydrogen peroxide for 8 min per round for four rounds significantly reduced the 24 h SBS between metal brackets and human teeth. Furthermore, although 10% sodium ascorbate followed by 37% phosphoric acid treatment increased the 24 h SBS compared with bleaching alone, the difference was not significant. Moreover, surface treatment with a one-step reagent composed of 35% 3-O-ethyl-l-ascorbic acid/50% citric acid for 5 min significantly restored the reduced bond strength compared with the bleaching group and was not significantly different compared with the unbleached teeth. Notably, the one-step surface treatment reagent reduced the treatment steps and clinical chair time compared with the 10% sodium ascorbate followed by 37% phosphoric acid treatment.

The adhesive remnant index (ARI) score revealed differences among the groups. The bleaching group without surface treatment had the highest frequency of failure at the tooth surface and adhesive interface, in contrast with the control group, where the majority of failures occurred in the adhesive layer. The surface-treatment groups with 10% sodium ascorbate and 35% 3-O-ethyl-l-ascorbic acid/50% citric acid reagent had a mixed pattern of failure at the interface between the enamel and adhesive and within the adhesive layer.

Overall, our results suggest that surface treatment with a one-step reagent composed of 35% 3-O-ethyl-l-ascorbic acid/50% citric acid for 5 min is a promising option for restoring the bond strength of teeth after bleaching and may be a more efficient alternative to traditional treatments, such as 10% sodium ascorbate followed by 37% phosphoric acid. However, further studies are needed to confirm our results and explore the optimal application protocols of the one-step surface-treatment reagent in the clinical setting.

## Figures and Tables

**Table 1:** Tooth specimen groups and their pre-bonding treatments.

Group	Number of Tooth Specimens	Tooth Bleaching Method	Surface Treatment	Etching Procedure
Control	10	None	None	37% PA ^1^
A	10	35% HP ^2^	None	37% PA
B	10	35% HP	10SA ^3^ 10 min	37% PA
C	10	35% HP	35EA/50CA ^4^ 5 min	None

^1^ PA: phosphoric acid; ^2^ HP: hydrogen peroxide; ^3^ 10SA: 10% sodium ascorbate; ^4^ 35EA/50CA: 35% 3-O-ethyl-l-ascorbic acid and 50% citric acid solutions.

**Table 2:** The mean and standard deviation of the shear bond strength (MPa) for each treatment by group.

Group ^A^	Mean ^B^	SD	Minimum	Maximum	Compared with	*p*-Value
Control	19.81	4.15	10.36	24.12	group A	>0.001
					group B	>0.001
					group C	0.06
A	8.55	3.66	4.80	15.85	group B	0.109
					group C	>0.001
B	12.09	2.67	7.40	16.48	group C	0.082
C	15.84	2.87	9.92	20.32		

^A^ Control = 37% phosphoric acid on unbleached teeth for 15 s; A = 37% phosphoric acid on bleached teeth for 15 s; B = 10% sodium ascorbate treatment for 10 min on bleached teeth, followed by 37% phosphoric acid for 15 s; C = 35%3-O-ethyl-l-ascorbic acid plus 50% citric acid solution on bleached teeth for 5 min. ^B^ The ANOVA test indicated that there were significant differences among the groups (*p* < 0.001).

**Table 3:** Adhesive layer fracture pattern after bracket debonding.

Groups *					
ARI Scores **	Control	A	B	C	Totals
0	2	9	7	5	23
1	3	1	2	4	10
2	4	0	1	1	6
3	1	0	0	0	1
Total	10	10	10	10	40

* The chi-squared test revealed significant differences among the groups (*p* < 0.01). ** ARI scores: 0 = no adhesive left on tooth’s surface; failure between adhesive and enamel; 1 = less than half of adhesive (<50%) left on tooth’s surface, 2 = half or more adhesive (>50%) left on tooth’s surface, 3 = all adhesive left on tooth’s surface; failure between adhesive and bracket base.

## Data Availability

Data are contained within the article.
